# Identifying Design Requirements for an Interactive Physiotherapy Dashboard With Decision Support for Clinical Movement Analysis of Musicians With Musculoskeletal Problems: Qualitative User Research Study

**DOI:** 10.2196/65029

**Published:** 2025-07-16

**Authors:** Eduard Wolf, Karsten Morisse, Sven Meister

**Affiliations:** 1 Health Informatics, Faculty of Health, School of Medicine, Witten/Herdecke University, Witten, Germany; 2 Healthcare Department, Fraunhofer Institute for Software and Systems Engineering, Speicherstraße 6, Dortmund, 44147, Germany, 49 231-97677 ext 450; 3 Department of Electrical Engineering and Computer Science, Faculty of Engineering and Computer Science, Osnabrück University of Applied Sciences, Osnabrück, Germany

**Keywords:** physical therapy, clinical movement analysis, clinical decision support, performance-related musculoskeletal disorders, requirements engineering, human factors engineering, design study methodology, problem-driven research, user-centered design, decision-centered design

## Abstract

**Background:**

Performance-related musculoskeletal disorders are common among musicians, requiring precise diagnostic and therapeutic approaches. Physiotherapists face unique challenges due to the complex relationship between musculoskeletal health and the demands of musical performance. Traditional methods often lack the necessary precision for this specialized field. Integrating clinical movement analysis (CMA) with clinical decision support (CDS) could improve diagnostic accuracy and therapeutic outcomes by offering detailed biomechanical insights and facilitating data-driven decision-making.

**Objective:**

This study aimed to identify design requirements for an interactive dashboard that aids clinical decision-making by incorporating CMA to assist physiotherapists in managing musculoskeletal disorders in musicians.

**Methods:**

A qualitative user research study was conducted, using human factors engineering methods from problem-driven research, user-centered design, and decision-centered design. Data collection included a domain-specific literature review, workflow observations, and focus group discussions with domain experts, including 4 physiotherapist experts and an expert for clinical reasoning and applied biomechanics. This qualitative data was triangulated to characterize the domain, identify the CMA workflow, user needs, key cognitive tasks, and decision requirements. These insights were translated into concrete design requirements.

**Results:**

A workflow for integrating musician-specific CMA into physiotherapy was established. In total, 21 user requirements, 7 key cognitive tasks, and 5 key decision requirements were defined, along with 49 design seeds. Key features identified include (1) efficient integration of musician-specific biomechanical findings into therapy, (2) combining heterogeneous data types for holistic assessment, (3) providing an adaptive overview of patient-related information, (4) using adequate visual representations and interaction techniques, (5) facilitating efficient visual-interactive analysis of findings and treatment results, and (6) enabling preparation and export of therapy findings and analysis results. Additionally, 14 CDS recommendations and 11 technical prerequisites were identified. These requirements guide the design of an interactive tool featuring advanced visualization, interactive data exploration capabilities, and contextual integration of clinical and biomechanical data.

**Conclusions:**

An interactive physiotherapy dashboard with CDS incorporating CMA data holds significant potential to enhance decision-making in physiotherapy for musicians with performance-related musculoskeletal disorders. By addressing cognitive demands and integrating advanced visualization techniques, the tool can support physiotherapists in making more accurate assessments, potentially improving patient outcomes, reducing injury recurrence, and supporting musicians’ career longevity. Ongoing research is essential to refine such a tool and validate its usability, decision support, and clinical effectiveness. Future work should explore advanced analytics, adapt to various CMA systems, and expand applications across musicians and therapeutic domains to enhance its impact.

## Introduction

### Background

Musculoskeletal disorders are common among musicians due to repetitive movements, muscle strain, poor posture, and intensive practice, affecting their ability to perform and sustain careers [1-3]. At least 50% of musicians experience performance-related musculoskeletal disorders (PRMDs), which cause pain and other symptoms that hinder performance [4-6]. However, targeted therapy is underused, and specialized assessment and treatment strategies are needed [7,8].

Musician-specific physiotherapy is beneficial for identifying and treating these issues, focusing on aspects of posture, movement behavior, and musical expression that affect health [8-10]. Effective treatment requires a thorough understanding of musician-specific characteristics and identification of functional disorders in the musculoskeletal system through clinical neuro-orthopedic examinations and analyses of the musician’s movement while performing [11].

In physiotherapy, clinical reasoning is essential for decision-making, often using a hypothetical-deductive method [12]. Accurate diagnostics are crucial to prevent misjudgments [13], and comprehensive information gathering is vital for effective therapy planning [14,15]. Technology-driven assessments can enhance diagnostic accuracy, moving beyond subjective methods and trial-and-error approaches [16].

Clinical movement analysis (CMA) provides objective, quantifiable data on functional disorders, supporting personalized medicine [11,17] and offering physiotherapy applications that conventional assessments cannot achieve [18,19]. As an apparatus-based biomechanical analysis for individualized functional diagnostics, CMA uses techniques such as motion capture and electromyography [20,21]. It helps evaluate complex movements during performance, allowing for the identification of aberrant postures and movements contributing to PRMDs in musicians [22-24]. Thus, CMA aids in diagnosing functional disorders, planning individualized therapy, and monitoring therapeutic measures [17,19,25], minimizing the risk of false results [26,27].

The *RefLabPerform* project [28] aims to develop a reference laboratory for PRMD assessment in musicians, integrating CMA with physiotherapeutic assessments, which will be implemented at the Institute for Applied Physiotherapy Osnabrück (INAPO). Advanced instruments will be used by specialists for musician-specific CMAs, with results provided to physiotherapists for further clinical evaluation.

However, interpreting CMA data requires expertise and time, presenting challenges for clinical practice, highlighting the need for efficient, intuitive presentation for analysis and decision-making [11,29,30]. Current tools for biomechanical analysis lack guidance for musicians’ physiotherapy, leading to concerns about accuracy and usability [30,31].

A tailored health technology for musicians’ physiotherapy is essential to efficiently record, process, provide, analyze, and interpret musician-specific CMA data for routine use. Hereby, an interactive dashboard with clinical decision support (CDS) can improve diagnostics by integrating CMA findings, facilitating comprehensive decision-making and effective therapies [32]. Adapting research approaches from health while considering physiotherapy-specific conditions will ensure the tool aligns with the clinical workflow, balancing perceived benefit, effort, and costs [16,33].

### Human Factors and CDS Design

Human factors engineering (HFE) is crucial for designing interactive health technologies and CDS tools, focusing on usability and workflow integration for decision support [34-36]. Effective CDS requires speed, efficiency, meaningful alerts, consistency, and logical grouping with intuitive interface design [37-40]. Hence, designing such a technology must address user needs, working environments, cognitive demands, decision-making requirements, and advanced interactive visualizations for data analysis.

In total, 3 HFE approaches—design study methodology (DSM), user-centered design (UCD), and decision-centered design (DCD)—provide guidance for this. DSM is a problem-driven research paradigm focused on creating innovative visualization solutions through iterative collaboration with domain experts to address real-world problems [41]. UCD emphasizes understanding user tasks, goals, and needs by involving users from the beginning, which informs the specification of user requirements and guides the design process [42]. DCD aims to improve decision-making in critical scenarios, such as medical diagnosis, by enhancing human decision-making [43,44]. Cognitive task analysis identifies key cognitive demands and translates them into decision requirements, guiding the design process to improve clinical performance [45]. These approaches share a human-centered, iterative, and multistage methodology, leveraging in-depth analysis and expert contributions, while each addresses distinct aspects of the design process.

### Objectives

The study aimed to identify design requirements for an interactive tool, specifically a physiotherapist dashboard, that aids clinical decision-making for musculoskeletal problems in musicians within the *RefLabPerform* project. This involved considering the working environment, clinical procedures, and user needs from the beginning and gathering detailed information on how decision-making in musicians’ physiotherapy can be supported technically, especially with the integration of CMAs.

To achieve this, requirements elicitation methods from DSM, UCD, and DCD were followed to comprehensively describe the domain and the underlying problem, resulting in well-informed requirements and recommendations. This approach is expected to enhance usability and acceptance, improve user efficiency and accuracy, and reduce the cognitive workload of the future tool.

## Methods

### Research Approach

**Figure 1.**
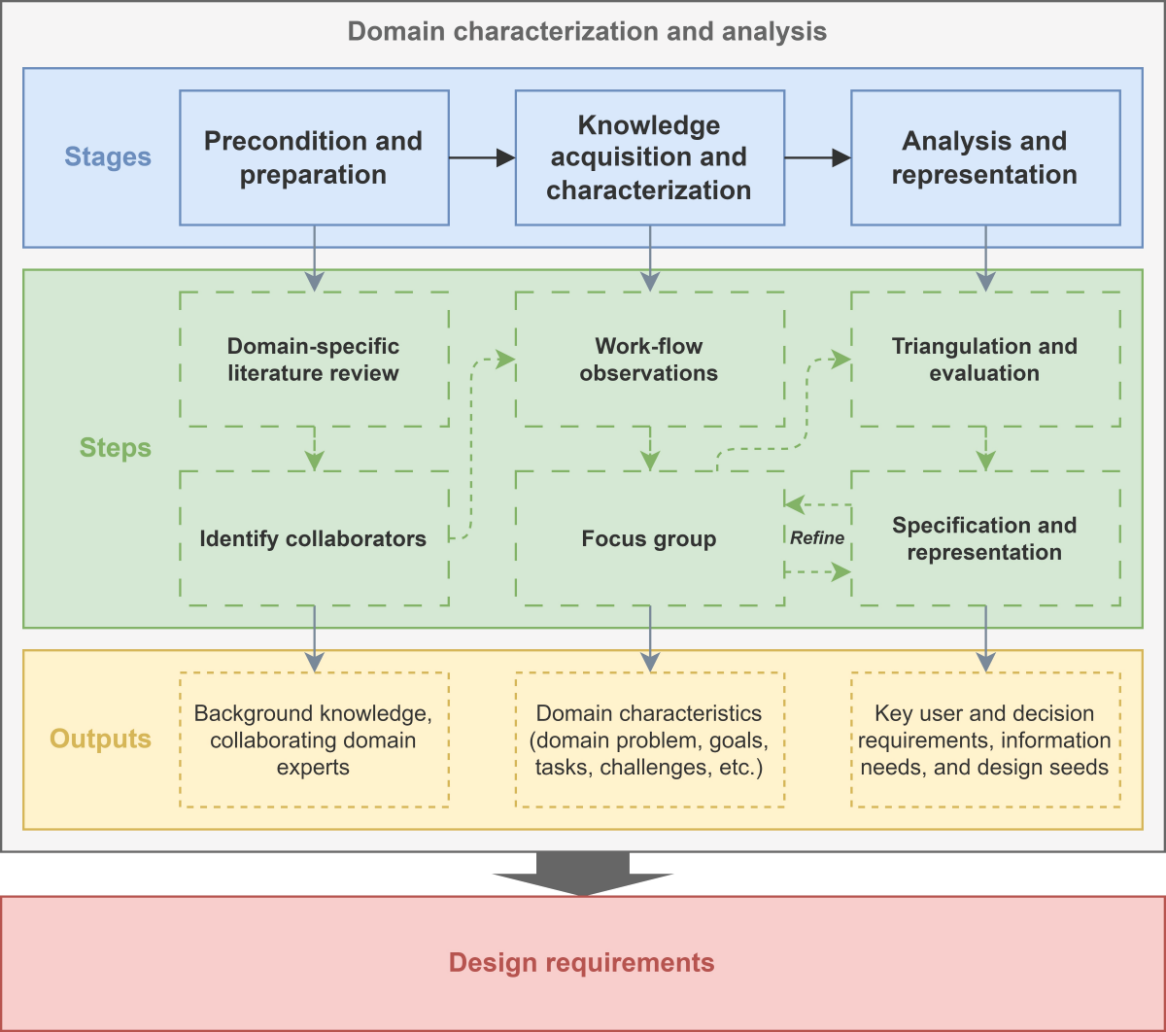
The domain characterization and analysis process comprised 3 stages with multiple steps and outputs, ultimately defining design requirements for an interactive physiotherapy dashboard.

This qualitative user research study followed a problem-driven research paradigm and formed part of the initial *domain characterization and analysis* phase within a larger design study [46]. It systematically integrated elements from DSM, UCD, and DCD to elicit and structure design requirements for an interactive dashboard with CDS functionality in physiotherapy (see Figure 1). The requirements elicitation process drew on the first 4 steps of DSM (*learn*, *winnow*, *cast*, and *discover*) [41], the initial 2 phases of UCD (*understanding context of use* and *specifying user requirements*) [42], and the first 3 stages of DCD (*preparation*, *knowledge elicitation*, and *analysis and representation*) [44]. These approaches were merged and operationalized into three stages: (1) precondition and preparation, (2) knowledge acquisition and characterization, and (3) analysis and representation, with subsequent design and evaluation phases planned for future work. DSM ensured methodological rigor and problem characterization through iterative domain expert engagement; UCD emphasized contextual inquiry and stakeholder needs; and DCD supported the analysis of complex cognitive processes in physiotherapy decision-making. Together, they provided a robust foundation for deriving clinically relevant, user-informed, and cognitively aligned design requirements. A detailed mapping of their contributions is presented in Table 1.

**Table 1.** Detailed mapping of each method’s contributions to domain characterization and analysis.

Research stage	Design study methodology (DSM)	User-centered design (UCD)	Decision-centered design (DCD)
Precondition and preparation	Domain understanding Relevant terms and jargon Identification of collaborators	Understanding user group characteristics and context of use	Identification of cognitively challenging activities from previous task descriptions
Knowledge acquisition and characterization	Focus on real-world domain problem characterization Domain expert engagement	Participatory engagement through workflow observations and focus groups Elicitation of user needs Focus on user goals and task alignment	Identification of cognitive demands, critical cues, and information needs
Analysis and representation	Iterative synthesis of findings into design seeds Structured representation of design implications	Specifying user requirements Integration of usability concerns Clarity and traceability of user needs to system design	Specifying decision requirements Integration of cognitive support needs in clinical workflow Translation of decision-making needs into design recommendations

The main author (EW) conducted the workflow observations and moderated the focus groups in German in March and April 2021. His researcher characteristics are the following: “gender: male,” “experience: 6 years research experience in health informatics, main focus in physiotherapy and movement analysis projects,” “degree: M.Sc. in Computer Science,” and “occupation: Research assistant.”

### Ethical Considerations

The study was conducted in accordance with the Declaration of Helsinki and approved by the institutional review board (or Ethics Committee) of Osnabrueck University of Applied Sciences (protocol code HSOS/2021/1/1, date of approval March 17, 2021). Signed informed consent for publication was obtained from all participants after providing them with a written information sheet and verbal explanation of the study context, the intended procedure, and data usage. All participants were given a copy of the signed informed consent as well as confirmation of anonymization of study data. The study data were anonymized, so participants cannot be identified. Participants did not receive any financial compensation for their participation.

### Precondition and Preparation

#### Domain-Specific Literature Review

Domain-specific literature from physiotherapy, clinical biomechanics, and movement analysis of musicians was reviewed to gather background information about the domain and user group characteristics and to become familiar with relevant terms and jargon [6,8-11,13-15,17,19,21,23,29,31,47-58]. Previously articulated task descriptions, such as clinical reasoning and functional diagnostics, were examined to gain domain understanding and to initially identify cognitively complex components.

#### Identify Collaborators

The INAPO, a specialized facility for treating neuromusculoskeletal disorders, served as a cooperation partner. INAPO has specialized in the treatment of musicians in close cooperation with the “Institute for Music” at Osnabrück University of Applied Sciences. Admitted patients receive tailored treatment and exercise programs. INAPO emphasizes staying updated with scientific progress in musicians’ health and movement sciences, using modern therapy methods. As part of the *RefLabPerform* project, a CMA lab was established at INAPO, equipped with optoelectronic motion capture, surface electromyography sensors, and force plates. Requirements were gathered based on the CMA lab and INAPO’s clinical practice needs.

In total, 4 physiotherapists (1 male, 3 female) from INAPO and an expert in clinical reasoning and applied biomechanics from the University of Applied Sciences Osnabruck were selected face-to-face as domain experts for systematic collaboration during requirements elicitation. Purposive sampling ensured the inclusion of a representative sample of practicing physiotherapists with extensive experience in managing musicians with musculoskeletal problems.

### Knowledge Acquisition and Characterization

#### Workflow Observations

We used contextual inquiry to observe the workflow of physiotherapists managing musicians with musculoskeletal problems at INAPO prior to the focus group discussion. Contextual inquiry, a qualitative user research method, involves observing and interviewing potential users in their work environment as they perform actual tasks [59], ie, watching them perform tasks while asking questions about their actions and thought processes. This method uncovers tacit knowledge, reveals detailed work practices, and provides reliable, relevant information about user needs and behaviors. The goal was to identify and gather domain experts' needs and preferences for a physiotherapist dashboard and CDS concerning their (cognitively challenging) tasks and daily data encounters. Additionally, we asked the experts about their thoughts on the integration of musician-specific CMAs into the diagnostic process and the application in clinical decision-making.

EW observed 3 physiotherapists experienced in managing musicians with musculoskeletal problems, previously selected for collaboration, during their daily work routines over 1 week. Each session lasted about 2 hours, allowing for detailed observation and discussion. Observations continued until thematic saturation was reached. Workflow diagrams were created from observation notes and presented at the focus group meeting to facilitate discussion on workflow processes.

#### Focus Group

We conducted a web-based focus group [60] lasting approximately 270 minutes (4.5 hours), comprising 7 participants: the 4 collaborating domain experts, 2 computer scientists, and 1 user experience designer. Video and audio recordings were made using Zoom (Zoom Video Communications, Inc). The focus group aimed to gain a deeper understanding of the domain, exploring the working environment, clinical procedures, user needs and decision-making processes in musicians’ physiotherapy, providing essential information for the tool’s design. The primary goal was to establish a common understanding of the domain and address the following key discussion topics for the specific setting: goals, skills, and barriers in musicians’ physiotherapy; required collaborations, tools, and equipment; workflow structure and objectives of each step; information, tools, and activities per work step; and motion analysis lab workflow and physiotherapist interaction.

Given the lack of standardized procedures for focus group data collection and analysis, which depend on the research question and time frame, we opted to organize the results into a mind map during the discussion to facilitate consensus-building. Following the focus group session, the research team held a debriefing session to review the discussions and identify key points.

### Analysis and Representation

#### Triangulation and Evaluation

The qualitative data was triangulated and evaluated to determine how the tool can address the domain problem and user needs, and support decision-making. First, the insights from workflow observations and the focus group were organized and contextualized with domain-specific literature to understand and describe the domain characteristics. Further evaluation of the findings revealed key user needs, as well as cognitively challenging tasks, strategies, difficulties, and critical aspects in musicians’ physiotherapy.

#### Specification and Representation

Based on the prior findings, the solution’s characteristics were specified. First, a workflow for integrating musician-specific CMA was established and visualized. Key user requirements, cognitive tasks, decision requirements, and associated information needs were then derived and formulated. Subsequently, design seeds to support decision-making were created. These findings were organized in tabular form.

In a second web-based focus group discussion, which lasted approximately 180 minutes (3 hours), the identified domain characteristics, specified user and decision requirements, and design ideas were revisited with the same group of experts and researchers. During this session, these elements were discussed in detail and refined based on their feedback.

Finally, incorporating knowledge and conditions from the theoretical background and domain, concrete design requirements were specified, including key features, technical prerequisites, and decision support recommendations.

## Results

### Characteristics of the Domain

To describe the domain, the characteristics were categorized under the following themes: domain problem; goals, skills, and competences, work equipment, and barriers; and tasks, routines, and challenges.

#### Domain Problem

The unresolved domain problem is integrating musician-specific CMA into musicians’ physiotherapy and offering visual-interactive access to aggregated findings and treatment data for decision support.

Musicians’ physiotherapy, an integral component of “musicians’ medicine,” specializes in the assessment and treatment of neuromusculoskeletal disorders prevalent among musicians. This specialty uses standard physiotherapeutic tools along with musician-specific questionnaires and CMAs to enhance diagnostic precision and facilitate data-driven therapeutic decisions. Post intervention, the collection of relevant patient questionnaires and repeated CMAs is crucial for effective therapy evaluation. However, an efficient technical solution for the comprehensive integration and analysis of clinical and biomechanical data tailored to musicians’ physiotherapy remains absent. The primary beneficiaries of such a solution would be physiotherapists, with musicians gaining from improved, targeted therapies. Additionally, specialized physiotherapists or movement scientists trained in musician-specific CMAs would benefit from enhanced integration of CMA into the therapeutic process.

Documentation, typically on paper, should be electronic for comprehensive evaluation. Integrating CMAs adds complexity, requiring standardized data processing and effective communication between physiotherapists and movement labs. Current software for biomechanical analysis is not user-friendly for physiotherapists, lacking advanced visualization, interactive data exploration, and contextualization of clinical and biomechanical data. A new solution is needed to simplify data analysis and visualization for therapists, with requirements evolving during implementation.

#### Goals, Skills, Competences, Equipment, and Barriers

Textbox 1 summarizes the goals, skills, competences, work equipment, and (potential) barriers cited by domain experts. The primary goal of physiotherapists is to provide high-quality patient care to enhance or maintain sensorimotor self-determination. This requires addressing the patient’s main problems and goals efficiently to implement effective, targeted therapies. It is crucial to assess the effectiveness of therapeutic measures continuously, aligning interventions with the patient’s condition and goals. Achieving these outcomes demands extensive skills and competencies, particularly in CR, which is central to effective therapy [58].

Textbox 1. **Domain-specific goals, skills and competencies, work equipment, and barriers.**

**Goals:**

- Enhancing or maintaining sensorimotor self-determination
- Improving the main problem
- Addressing patient goals

**Skills and competencies:**

*Background knowledge:*

- Patho-anatomy and physiology (clinical patterns, clinical picture, and musician-specific traits)
- Reference values (standard, individual, and musician-specific)
- Scientific evidence for assessments, tests, interventions

*Clinical experience:*

- Communication skills
- Manual skills
- Recognizing clinical patterns
- Techniques
- Test interpretation

**Equipment:**

- Patient questionnaires
- Diagnostic questionnaires
- Body tables
- Numerical rating scale
- Measuring instruments (eg, goniometer, algometer)

**Barriers:**

*Physiotherapist:*

- Lack of background knowledge
- Lack of clinical experience
- Shift in knowledge and beliefs, eg, through further education and training
- Premature judgments

*Patient:*

- Compliance
- Background (cognitive, social, and educational)
- Negative experiences
- Unreasonable expectations

*General conditions:*

- Legal: Limited time, lack of resources (eg, financial)
- Institutional: Limited time, lack of resources (eg, premises, measuring instruments), availability of qualified therapists

Domain experts highlight the importance of a solid background in (patho-)anatomy, physiology, normative and comparative values, and evidence-based assessments, tests, and interventions. Clinical experience is also vital for developing communication and manual skills, refining techniques, interpreting tests, and recognizing clinical patterns. Further general requirements for physiotherapists are outlined in the literature [58]. Domain experts identified several tools used in therapy, including musician-specific patient questionnaires, body tables, numerical rating scales, goniometers, and algometers.

Physiotherapists face several barriers that can impede therapeutic objectives, including insufficient background knowledge, limited clinical experience, and shifts in knowledge due to ongoing education. Patient-related barriers include compliance issues and unrealistic expectations. Additionally, legal or institutional barriers, such as limited examination and treatment time, and insufficient financial, technical, or spatial resources, also pose significant challenges.

#### Tasks, Routines, and Challenges

The cyclical CR process consists of 8 main phases: looking, collecting, processing, deciding, planning, acting, evaluating, and reflecting [12]. These phases are interrelated and often overlap. The technical solution should explicitly assist the therapist in 5 of these steps (Textbox 2):

Textbox 2. **Five steps that a technical solution should assist the therapist.**

- **Collecting:** recording all subjective and objective findings
- **Processing:** analyzing and interpreting the findings
- **Deciding:** assessing the findings for diagnosis and planning
- **Evaluating:** assessing the interventions based on the repeat findings
- **Reflecting:** assessment of therapy effectiveness

The diagnostic process in physiotherapy involves examination, evaluation, and reassessment of findings and therapy measures under significant time pressure. Therapists must extract relevant information to make accurate treatment decisions, recognizing anomalies and changes in data [61].

Workflow observations and focus group discussions provided a deeper understanding of the therapeutic and diagnostic processes in musicians’ physiotherapy. Before the first consultation, patients’ complete musician- and problem-specific questionnaires, and the results guide the subjective examination, including background information and symptoms. This information helps generate diagnostic hypotheses tested during a physical examination, often using tools like goniometers. The results confirm or reject these hypotheses, leading to a physiotherapeutic diagnosis and a customized treatment plan. Treatment effectiveness is evaluated regularly, with reassessments before each follow-up to adjust or stop treatment as needed.

### Specification and Representation

The findings from the prior domain characterization were further examined and discussed with the domain experts. This process aimed to address the integration of musician-specific CMA into physiotherapy, identify user requirements, and determine key cognitive tasks and decision requirements.

#### Integrating Musician-Specific CMA Into Physiotherapy

Based on the domain experts’ input, the integration of musician-specific CMA into physiotherapy was designed. It is crucial to incorporate CMA efficiently, providing valuable information on a musician’s dysfunction. The timing, execution, and result delivery are critical for high clinical benefit and rapid transfer from the movement analysis lab to the therapist. The CMA aims to test diagnostic hypotheses and evaluate therapeutic measures’ effectiveness. Biomechanical data should be classified and interpreted within the clinical context, structured as additional findings, and made available to therapists.

**Figure 2.**
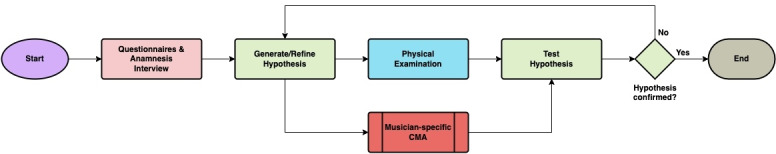
The revised diagnostic process integrates clinical movement analysis (CMA) as a complementary tool alongside physical examination (own illustration). It begins with musician and problem-specific questionnaires and anamnesis, followed by the generation of diagnostic hypotheses. A physical examination is then conducted to test these hypotheses. Simultaneously, a musician-specific CMA, guided by the hypotheses and a predefined movement analysis protocol, provides objective validation. If hypotheses require refinement or new ones emerge, additional physical exams and CMA assessments are performed until the final hypotheses are confirmed.

Figure 2 proposes integrating CMA into the assessment process alongside the physical examination. Clinical reasoning underpins CMA use, starting with a clear question based on a working hypothesis, guiding the selection of muscles, starting positions, testing types, and biomechanical characteristics. This procedure is referred to as a movement analysis protocol based on Kontaxis et al [62].

**Figure 3.**
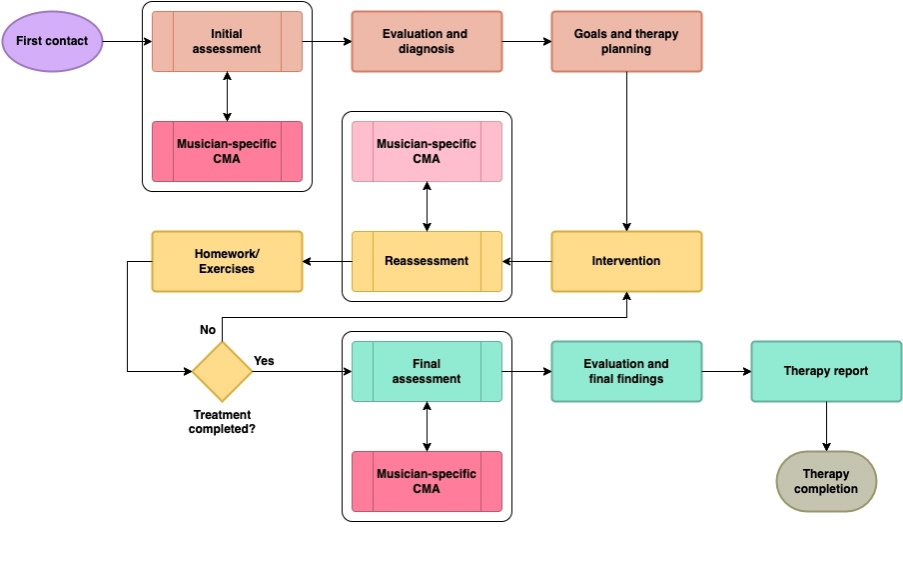
The modified clinical procedure integrates clinical movement analysis (CMA) for pre-, intra-, and posttreatment assessment (own illustration). Following the initial contact, an assessment is conducted, leading to findings evaluation, final diagnosis, goal setting, and therapy planning. After intervention, a reassessment is performed, and patient homework exercises are provided. If treatment continues, further interventions and reassessments follow; if completed, a final assessment, therapy evaluation, and report are conducted before concluding therapy.

In addition to using CMA for functional diagnostics during the initial assessment, a subsequent CMA is conducted post treatment, as depicted in Figure 3. This allows for a pre-post comparison to evaluate the effectiveness of the treatment interventions.

#### User Requirements

Contextual analysis, informed by the domain-specific literature, workflow observations, and focus groups, revealed user needs concerning the solution’s characteristics. In total, 21 user stories were defined based on these needs, reflecting the users’ expectations of the physiotherapist dashboard’s functionality [63]. These user stories pertain to clinical findings and treatment documentation, CMA, analysis and evaluation, and reporting. The full list of user requirements is available in Multimedia Appendix 1.

#### Cognitive Tasks and Decision Requirements

Cognitively challenging activities were identified, such as diagnostics, treatment planning, and therapy evaluation, informed by task descriptions from literature and the findings from knowledge acquisition and characterization. For each activity, we identified critical cues used by experts, potential errors, and procedures to meet cognitive demands. Physiotherapists generate and test hypotheses by interviewing patients and conducting or commissioning examinations, continuously integrating, and interpreting the information. Each activity is a cognitively challenging demand, requiring decisions on what information to gather and when to stop, as well as determining the next steps in treatment.

Finally, seven key cognitive tasks were identified: (1) querying specific patient questionnaires, (2) subjective examination of the patient, (3) testing diagnostic hypotheses, (4) consolidating and evaluating the information relevant to therapy, (5) planning the adapted therapy, (6) treating a patient’s specific problem in an iterative approach, and (7) evaluating the therapy results. The final list of key cognitive tasks and the associated strategies, (potential) errors or difficulties, and critical hints of the domain experts can be found in Multimedia Appendix 2.

In coordination with the domain experts, key decision requirements were extracted from these activities, focusing on planning and assessing examinations and treatments while considering musician-specific CMA. Information needs for each decision were defined, specifying the clinical information needed. This approach led to design recommendations for decision support, offering early human-computer interaction concepts for organizing and visualizing information.

In total, five key decision requirements were identified incorporating musician-specific CMA in the context of physiotherapeutic diagnostics: (1) plan a functional diagnostic examination; (2) assess CMA findings; (3) prepare overall findings, and, if necessary, a report of findings; (4) compare results of reassessments and evaluate treatment effectiveness; and (5) evaluate therapy results and success. Based on the key decision requirements and associated information needs, a total of 49 design seeds were identified. The final list can be found in Multimedia Appendix 3.

Physiotherapists must decide if, when, and how to perform a CMA for a patient, considering basic patient information, contributing factors, symptoms, previous CMA parameters, and time and number of previous treatments. They must quickly evaluate findings to plan next steps, gaining an overview of the patient’s problem, identifying patterns, and confirming diagnostic hypotheses to formulate a physiotherapy diagnosis. They must determine which data are therapy-relevant to create an overall finding or report.

Throughout treatment, physiotherapists continuously assess findings and treatment effectiveness, deciding if measures positively impact the patient’s problem or need adjustment. They review therapy-relevant information before and after treatments to identify trends and changes. They reflect on therapy outcomes and their actions, assessing whether therapy was successful and identifying key factors. They monitor achievement of therapy and patient goals using data from baseline and follow-up findings, treatment results, and relevant questionnaires, including post-intervention CMA results.

### Design Requirements

The *domain characterization and analysis* informed concrete design requirements for a physiotherapist dashboard, specifying key features and CDS recommendations based on user needs for decision support. The use case defined technical prerequisites for implementation.

#### Key Features

Based on the insights gained, we identified 6 key features (KF) that form the basic pillars of the tool and must be fulfilled during design and implementation:

- **KF_1_**. *Provision of biomechanical findings:* Musician-specific CMAs should be efficiently integrated into therapy to give physiotherapists valuable insights into a patient’s dysfunction. Biomechanical data must be processed, modeled, and stored to produce a report with relevant characteristics for further analysis.
- **KF_2_**. *Integration of heterogeneous data types and sources:* For a holistic inspection and assessment of all available information from the subjective and objective examinations of a patient’s problem, it is necessary to combine different types of data from different data sources. This includes clinical findings and treatment data from physiotherapy documentation, results from web-based questionnaires, and biomechanical findings data from movement analysis.
- **KF_3_**. *Adaptive overview:* A simplified aggregated overview is essential for quick and clear inspection and monitoring of patient-related information. This should include basic therapy data, recent findings, and treatments, as well as relevant individual patient details. Information should be adaptive and patient-specific, allowing physiotherapists to manually add or remove data. Detailed views should be accessible via a drill-down function.
- **KF_4_**. *Adequate visual representations and interaction techniques:* Efficient visualizations, such as lists, tables, diagrams, and illustrations, should be chosen to aid physiotherapists in effective decision-making. Therapists should be able to manually arrange and configure these elements and use familiar interaction techniques like drag-and-drop, sorting, searching, and filtering.
- **KF_5_**. *Efficient visual-interactive analysis of findings and treatment results:* Physiotherapists need an efficient solution for interactive visual analysis of findings and treatment results to identify abnormalities, trends, and patterns in clinical and biomechanical data. Established visual analytics methods will be used to explore and compare multivariate and time-oriented data.
- **KF_6_**. *Preparation and export of findings and analysis results:* Physiotherapists should be able to summarize and export therapy findings as a report for discussion with colleagues and patients, or for continued treatment elsewhere. The report should be presented in a clear, customizable format.

#### CDS Design Recommendations

The user needs and design seeds addressing decision requirements formed the basis for specifying concrete CDS design recommendations (DR; see Textbox 3).

For planning musician-based diagnostics, it is helpful to have a note indicating if such an examination is possible for the patient (DR_1_). CDS should remind the physiotherapist to conduct pre- and post-interventional exams if possible (DR_2_). Additionally, suggested configuration parameters should be provided when creating an examination order to save time (DR_3_).

To efficiently query and inspect findings and treatment results, essential data should be preselected for the overview (DR_4_). Information should be summarized in aggregated elements and presented in suitable display formats (DR_5_). Relevant therapy events should be shown on a timeline (DR_6_). Additionally, visual cues should highlight significant data changes to reduce cognitive load and speed up decision-making (DR_7_).

For interpreting findings, integrating normative and comparative values is desirable (DR_8_). Therapists should be able to create interactive annotations (DR_9_), marking specific data values or time intervals. To support diagnosis, findings and annotations should be linked to diagnostic hypotheses, showing which data support or refute them (DR_10_). Additionally, marking applicable or inapplicable hypotheses interactively should be possible (DR_11_).

For therapy evaluation, directly comparing baseline and final findings is essential (DR_12_). An interactive review of patient and therapy goals helps determine therapy success, requiring target parameters for goal achievement (DR_13_). Preselecting relevant entries and suitable presentation forms aids in report preparation (D_14_).

Textbox 3. **Overview of the clinical decision support design recommendations (DR).**

**Planning musician-specific examinations for functional diagnostics**

**DR_1_**: Indicate if musician-specific examinations are possible for the patient.

**DR_2_**: Remind physiotherapists to conduct pre- and postinterventional examinations.

**DR_3_**: Provide suggested configuration parameters for examination orders to save time.

**Query and inspection of diagnostic findings and treatment results**

**DR_4_**: Preselect essential data for efficient querying and inspection.

**DR_5_**: Summarize information in aggregated elements and suitable display formats.

**DR_6_**: Map relevant events on a timeline.

**DR_7_**: Use visual cues to highlight abnormal data or significant changes.

**Analysis and interpretation of the findings data**

**DR_8_**: Integrate normative and comparative values for interpreting findings.

**DR_9_**: Allow interactive annotations for specific data values or time intervals.

**DR_10_**: Link findings and annotations to working hypotheses.

**DR_11_**: Mark working hypotheses as applicable or not with appropriate presentation.

**Therapy evaluation and reporting**

**DR_12_**: Enable direct comparison of baseline and final findings for therapy evaluation.

**DR_13_**: Facilitate an interactive review of patient and therapy goals, with target parameters for goal achievement.

**DR_14_**: Preselect relevant entries and use appropriate presentation formats for report preparation.

#### Technical Prerequisites

Technical prerequisites (TP) must be established to enable the physiotherapist dashboard (see Textbox 4). The tool should allow physiotherapists to access patient data and case history (TP_1_), including anamnesis, physical exams, and treatment records (TP_2_). Patients must complete validated musician- and problem-related questionnaires web-based, with results integrated into the findings overview (TP_3_). Biomechanical data from musician-specific CMAs should be available as findings (TP_4_).

Functional diagnostics occur in a CMA lab within a physiotherapy practice, staffed by specialists. Physiotherapists must submit *electronic exam orders* with relevant CMA details (eg, patient ID, instrument group, and symptoms; TP_5_). The lab requires an *order management system* to track requests and update CMA status (TP_6_) and a *data management system* for recorded measurements (TP_7_). Standardized, musician-specific CMA protocols should be implemented (TP_8_), integrating existing measurement tools and software (TP_9_). Data processing and calculations should be largely automated (TP_10_), with results provided as biomechanical findings (TP_11_).

Textbox 4. **Overview of the technical prerequisites (TP) to enable the physiotherapist dashboard.**

**Technical prerequisites (TP)**

**Integration of therapy-related patient data**

**TP_1_**: Integration of patient and case data

**TP_2_**: Integration of clinical findings and progress data

**TP_3_**: Integration of the web-based questionnaire results

**TP_4_**: Integration of the biomechanical findings data

**Laboratory system**

**TP_5_**: Electronic order entry

**TP_6_**: Laboratory order management

**TP_7_**: Laboratory data management

**TP_8_**: Standardized motion analysis protocols

**TP_9_**: Integration of existing biomechanical measuring instruments or systems and analysis software

**TP_10_**: (Largely) automated processing and calculation of relevant features

**TP_11_**: Providing data from CMA as biomechanical findings

## Discussion

### Principal Findings

We proposed and applied a novel HFE process model to inform the design of a physiotherapist dashboard with CDS for CMA of musicians with musculoskeletal problems, using methods, and addressing aspects from DSM, UCD, and DCD. Following this model, we first identified and thoroughly described the characteristics of the domain and problem area. We explored user needs, cognitively challenging tasks, information needs, and decision-making challenges faced by physiotherapists managing musicians with musculoskeletal conditions. We then proposed design seeds for CDS, translating user and decision requirements into concrete design requirements, including key features, technical prerequisites, and recommendations for decision support.

Key features include efficient integration of biomechanical data, combining heterogeneous data types, and providing adaptive overviews for quick data inspection. The tool should present patient data in a suitable form and facilitate interactive visual analyses using familiar techniques like filtering, highlighting, and annotating. Summarizing and exporting findings for communication are also essential. User and decision requirements led to specific decision-support recommendations: CDS should indicate if musician-specific diagnostics are possible, remind physiotherapists of physical exams, suggest efficient configuration parameters, preselect essential data, highlight significant changes, integrate normative values, support interactive annotations, compare baseline and final findings, and provide tools for reviewing patient goals and preparing reports. To enable the tool, technical prerequisites must be met, including access to patient data (anamnesis, physical exams, treatments, and problem- or musician-related questionnaires), biomechanical data from musician-specific CMAs, support for electronic diagnostic orders, and management of these orders and CMA statuses. The tool should use standardized protocols, integrate with existing tools, and automate data processing to present biomechanical findings.

Additionally, our study contributes to the existing literature by detailing a methodology for requirements elicitation, comprehensively describing the domain of musicians’ physiotherapy, and identifying requirements for the successful use of the tool. Our findings support previous research indicating that HFE is crucial for designing CDS technologies [34,35], emphasizing aspects such as speed, efficiency, user needs anticipation, workflow integration, meaningful alerts, consistency, logical grouping, interface design, and design simplicity [37-40].

### Limitations

This study is in the early stages of design, focusing on establishing a structured foundation for the tool’s design and domain-specific clinical workflows, relying primarily on qualitative methods like workflow observations and focus group discussions. While the findings provided in-depth qualitative insights from a small group of domain experts, generalizability is limited, and broader validation is needed. To address this, we plan to conduct a Delphi study involving a larger and more diverse panel of physiotherapists, movement scientists, and other relevant stakeholders [64]. This structured, iterative process will facilitate expert consensus on the tool’s design requirements, ensuring their robustness and applicability across various physiotherapy contexts. The Delphi study will also help refine the identified requirements based on collective expert feedback, enhancing the tool’s validity before further prototyping, usability testing, and implementation in clinical practice.

At this stage, no functional prototype exists, and technical features or implementation details, such as specific visualizations, interaction methods, and decision logic, were not addressed. These aspects will be defined and iteratively refined during future stages of prototyping and evaluation. Furthermore, quantitative evaluation of metrics such as diagnostic accuracy, efficiency, or patient outcomes is not yet feasible. Future work will involve the development of a prototype, followed by iterative usability testing and structured evaluations. These evaluations will incorporate quantitative measures—such as time-to-decision, task completion rates, diagnostic decision support effectiveness, efficiency gains, and user satisfaction scores—to assess the tool’s impact and clinical utility in real-world physiotherapy settings.

In addition, artificial intelligence (AI)–based CDS features were overlooked. Integrating machine learning and predictive analytics could significantly enhance precision and relevance [65]. In future development stages, machine learning algorithms can be incorporated to support early diagnosis, pattern recognition, as well as personalized treatment planning and outcome prediction considering individual patient characteristics. For example, supervised learning models could be trained on annotated CMA data to automatically identify movement anomalies or classify injury risks [65]. Predictive analytics could also be used to forecast recovery progression based on patient-specific parameters such as movement patterns, therapy progress, and contextual factors (eg, instrument type or workload) [66]. These enhancements would augment the therapist’s decision-making with advanced data-driven insights while maintaining transparency and clinician control. Overall, this highlights the tool’s extensibility and long-term innovation potential.

Moreover, while the proposed CDS approach focuses on integrating CMA with gold-standard methods, AI-driven approaches like computer vision-based pose estimation (eg, OpenPose [67]) should be explored. Sensorless systems could provide nonintrusive, real-time movement tracking, making assessments more accessible, especially in nonlaboratory settings. AI pattern recognition could automate impairment classification and suggest diagnostic hypotheses, aiding physiotherapists and improving decision-making. It could also enable remote physiotherapy, allowing musicians to exercise at home with real-time feedback. However, challenges in accuracy, occlusion, and clinical validation must be addressed before full integration into physiotherapy workflows.

### Opportunities and Challenges

The integration of CMA into physiotherapy and incorporating clinical and biomechanical data with CDS within an interactive dashboard present both opportunities and challenges. Our study emphasizes the need to tailor CDS to real clinical environments and therapists’ workflows. Ensuring these technologies are perceived as useful is crucial, as the balance between perceived benefits and implementation effort significantly affects adoption. Developing an interactive tool for supporting diagnosis and treatment of musculoskeletal disorders in musicians requires a multidisciplinary approach, combining health informatics, biomechanics, and clinical practice. Collaboration with domain experts ensures the tool meets practical needs. Requirements were elicited through problem-driven research, ensuring that real-world problems were addressed, as well as UCD and DCD approaches, considering user needs, cognitive demands, and decision requirements, aiming to enhance the usability and usefulness of the solution.

Most existing tools in physiotherapy focus on general clinical documentation [68] or basic measurement instruments like goniometers and numeric rating scales [18], with limited quantitative and objective significance. Advanced biomechanical analysis systems (eg, Qualisys [69]) remain primarily research tools—technically complex and disconnected from everyday clinical decision-making, lacking user-friendliness, tailored visualizations, and integration with clinical reasoning. CDS tools in physiotherapy are still emerging, with AI-based approaches showing promise for personalizing treatment and predicting patient-specific outcomes [70-72]. However, they lack integration of CMA into clinical workflows and provide limited support for context-specific decision-making, particularly for niche populations like musicians with PRMDs. Existing visualization tools support rehabilitation or monitoring but do not incorporate CMA data [73,74], and those that do focus on clinical gait analysis or specific disorders [75,76]. Currently, no tool integrates CMA data into the decision-making process for physiotherapists managing musicians with PRMDs.

An interactive tool based on the identified design requirements would fill this gap by directly integrating CMA into clinical workflows, aligning with physiotherapists’ reasoning. It would enable interactive visualizations tailored to the needs of diagnosing and treating musicians with PRMDs, offering flexibility for different instruments and playing styles. Unlike existing tools, it bridges the gap between biomechanical analysis and clinical practice, providing musician-focused support and a platform for integrating advanced analytics to enhance diagnostic precision and longitudinal tracking. Such a system aims to integrate CMA into real-world physiotherapy workflows, providing context-aware support and decision facilitation across all therapy stages, particularly for underserved populations like performing artists.

The successful deployment of a future CDS tool will require addressing key technical and ethical challenges. Integration into existing health care IT systems involves overcoming data heterogeneity, ensuring interoperability, safeguarding data privacy, and achieving clinician adoption. Many institutions rely on proprietary electronic health records [77], complicating seamless data exchange. Standardized protocols such as HL7 (Health Level Seven) and FHIR (Fast Healthcare Interoperability Resources) can help address interoperability [78], while compliance with regulations like GDPR (General Data Protection Regulation) will require robust encryption, anonymization when applicable, and role-based access controls. Diverse IT environments further necessitate system compatibility; a modular, cloud-based architecture could offer the flexibility and scalability needed for broad adoption [79]. Clinician adoption will depend on smooth integration into existing workflows, user-friendly interfaces, and comprehensive training [80].

Future work must standardize and integrate diverse data sources—such as motion capture outputs, clinical notes, and patient records—into a unified platform compatible with physiotherapy workflows. Ethical considerations are equally critical. The envisioned tool is designed to assist, not replace, physiotherapists by providing objective biomechanical data to enhance—not automate—clinical decision-making. The system will maintain clinician autonomy, ensure transparency and interpretability, and be developed in adherence to data protection regulations. Although risks related to automation or bias are minimal at this stage, they will remain important considerations in later development phases, particularly as data handling, user control, and AI components evolve. Finally, the evolving nature of clinical practices and data types presents ongoing challenges for maintaining a CDS tool’s relevance. Continuous updates are needed to keep pace with advancements in clinical knowledge and technology, which can be resource-intensive. Addressing these challenges in future research will enhance such a tool’s usability, adaptability, and impact on clinical decision-making.

In conclusion, successful implementation of the physiotherapist dashboard with CDS based on musician-specific CMA depends on alignment with clinical workflows, ease of use, successful integration in existing IT infrastructure, maintaining data protection and clinician autonomy, and demonstrable benefits in diagnostic accuracy and treatment efficiency. Insights from this study provide a solid foundation for designing an interactive tool for physiotherapists, incorporating CMA for musicians with musculoskeletal demands, aiming to improve patient outcomes and support clinical decision-making. Iterative feedback from domain experts during design and development should ensure the tool addresses real-world problems, is validated through practical application, and is continuously refined based on user insights. The involvement of physiotherapists and movement scientists from initial design to validation is pivotal.

While this study focuses on the niche context of musicians with PRMDs, the identified design requirements, domain-specific decision-making insights, and integration of CMA hold potential for broader application. The underlying framework and methodology can be adapted to other physiotherapy contexts that involve complex, movement-related diagnostic and therapeutic challenges—such as in athletes, dancers, or individuals with work-related repetitive strain or posture-related injuries. These populations similarly require tailored functional assessments and context-specific CDS [81-84]. Modular, task-oriented design supports adaptation across these groups by enabling customization of movement analysis protocols, clinical decision parameters, and visualization formats to meet domain-specific needs. This enhances both the generalizability and scalability of the proposed framework. Future work may explore how the proposed process model and design requirements can be transferred and tailored to other physiotherapy contexts, extending the broader relevance and impact of this research beyond musicians.

### Conclusion and Future Work

This work contributes to HFE, physiotherapy, CMA, and CDS for musicians with PRMDs by combining rigorous research methods with expert insights. The proposed process model helps stakeholders identify user needs and decision-making requirements at the point of care, while the design requirements lay the groundwork for developing a specialized interactive physiotherapist dashboard integrating CMA with CDS. This tool can enhance diagnostic accuracy and therapy effectiveness through interactive, visual data presentation, streamline workflows, and maintain clinical relevance. Ultimately, it aims to improve patient outcomes, reduce injury recurrence, and support musicians’ long-term careers.

In the next phase, we plan to translate the design requirements into a functional prototype as part of a physiotherapy-integrated diagnostic system [85]. To ensure practicality and effectiveness, we will conduct rigorous usability testing and evaluations with physiotherapists. This will involve iterative feedback loops through think-aloud protocols and task-based evaluations, ensuring that the tool aligns with clinical workflows and cognitive demands. Structured feedback will be used to refine interface features, data presentation, and interaction flows, providing empirical evidence of feasibility, usability, and alignment with the identified requirements.

Future research should validate usability, decision support, and clinical effectiveness; explore advanced analytics; adapt to various CMA systems (eg, sensorless tracking); and expand applications across diverse musicians, instruments, and therapeutic domains to enhance its impact.

## Supplementary material

10.2196/65029 Multimedia Appendix 1 User requirements (UR) formulated as user stories based on user needs for decision support.

10.2196/65029 Multimedia Appendix 2 Cognitive tasks and associated strategies, (potential) errors or difficulties, and critical hints.

10.2196/65029 Multimedia Appendix 3 Key decision requirements (DR), associated information needs and design seeds (S).

## Abbreviations

AI: artificial intelligence
CDS: clinical decision support
CMA: clinical movement analysis
DCD: decision-centered design
DSM: design study methodology
FHIR: Fast Healthcare Interoperability Resources
GDPR: General Data Protection Regulation
HFE: human factors engineering
HL7: Health Level Seven
INAPO: Institute for Applied Physiotherapy Osnabrück
PRMD: performance-related musculoskeletal disorder
USD: user-centered design
