# Supplementary material for: Identifying Design Requirements for an Interactive Physiotherapy Dashboard With Decision Support for Clinical Movement Analysis of Musicians With Musculoskeletal Problems: Qualitative User Research Study
Source: JMIR Hum Factors. 2025 Jul 16;12:e65029. doi: 10.2196/65029 (PMC12292032; doi:10.2196/65029)
Supplement: Multimedia Appendix 1 [file humanfactors-v12-e65029-s001.docx]

# Multimedia Appendix 1: User requirements (UR) formulated as user stories based on user needs for decision support.

| **Category** | **User Requirement** | |
| --- | --- | --- |
| Clinical findings and treatment documentation | **U_R1_** | As a *physiotherapist*, I want to be able to view the outcomes of instrument- and problem-specific online questionnaires, so that I can check a patient's subjective state of health. |
|  | **U_R2_** | As a *physiotherapist*, I want to be able to view the outcomes of the medical history, so that I can check a patient's subjective self-assessment. |
|  | **U_R3_** | As a *physiotherapist*, I want to be able to view the outcomes of the physical examination, so that I can check the objective information on a patient's problems. |
|  | **U_R4_** | As a *physiotherapist*, I want to be able to view the interventions carried out, so that I can follow the course of treatment. |
|  | **U_R5_** | As a *physiotherapist*, I want to be able to view the therapy and patient goals, so that I can check these after the treatment has been completed. |
| Clinical movement analysis | **U_R6_** | As a *physiotherapist*, I want to be able to order a standardized and musician-specific CMA, so that I can obtain reliable and objective information about an instrumentalist’s playing movement. |
|  | **U_R7_** | As a *physiotherapist*, I want to be able to set examination parameters based working hypotheses, so that I can target the examination and confirm or reject the hypotheses. |
|  | **U_R8_** | As a *physiotherapist*, I want to be able to view the status of an instrumentalist’s CMA, so that I can check the progress. |
|  | **U_R9_** | As a *physiotherapist*, I want to be able to retrieve relevant outcomes of an instrumentalist’s CMA, so that I can review the biomechanical data on an instrumentalist's playing movement. |
|  | **U_R10_** | As a *physiotherapist*, I want to be able to view biomechanical findings in a temporally aligned way, so that I can inspect the data from different measuring systems in a in a holistic way. |
|  | **U_R11_** | As a *physiotherapist*, I want to be able to view activities from the CMA in a temporally aligned way, so that I can compare intra-individual findings of different measurement times. |
|  | **U_R12_** | As a physiotherapist, I want to be able to order additional standardized and musician-specific CMA, so that I can investigate intervention-related changes. |
| Analysis and evaluation | **U_R13_** | As a *physiotherapist*, I want to be able to obtain a quick overview of all therapy events, so that I can efficiently understand the entire course of therapy for a patient. |
|  | **U_R14_** | As a *physiotherapist*, I want to be able to obtain a quick overview of all patient-related findings, so that I can check the current state of health of a patient. |
|  | **U_R15_** | As a *physiotherapist*, I want to be able to have the patient-related findings and treatment results represented in such a way that I can assess them efficiently. |
|  | **U_R16_** | As a *physiotherapist*, I want to analyze the findings in detail, so that I can arrive at a comprehensive understanding of a patient's problem. |
|  | **U_R17_** | As a *physiotherapist*, I want to confirm, adapt, or reject the current (working) hypotheses based on the findings, so that I can arrive at a physiotherapeutic diagnosis of a patient's problem. |
|  | **U_R18_** | As a *physiotherapist*, I want to extract the most important information, so that I can summarize it into an overall finding. |
|  | **U_R19_** | As a physiotherapist, I want to compare the final finding with the basic finding, so that I can finally check the success and effect of the therapy. |
| Reporting | **U_R20_** | As a *physiotherapist*, I want to be able to create a report with the relevant information, so that I can enable the patient to continue treatment elsewhere. |
|  | **U_R21_** | As a *physiotherapist*, I want to be able to create a therapy report, so that I can summarize the progress and therapy results of a patient and, if necessary, to send it to a physician. |
