# Supplementary material for: Identifying Design Requirements for an Interactive Physiotherapy Dashboard With Decision Support for Clinical Movement Analysis of Musicians With Musculoskeletal Problems: Qualitative User Research Study
Source: JMIR Hum Factors. 2025 Jul 16;12:e65029. doi: 10.2196/65029 (PMC12292032; doi:10.2196/65029)
Supplement: Multimedia Appendix 2 [file humanfactors-v12-e65029-s002.docx]

# Multimedia Appendix 2: Cognitive tasks and associated strategies, (potential) errors or difficulties, and critical hints.

| **Cognitive TASK** | **Strategy** | **Error/Difficulty** | **Critical Hints** |
| --- | --- | --- | --- |
| 1. Querying specific patient questionnaires | - Physiotherapist asks for the patient's self-assessment using specific questionnaires. - Patient responds to the questionnaire items. | - Patient provides incomplete or untruthful answers. | - Psychosocial factors - Musician-specific factors - Dysfunction-dependent factors - Pain mechanism and localization - Total and sub-scores |
| - 1. Deciding which questionnaires to give to the patient | - Physiotherapist selects questionnaires based on patient information and the specific problem. | - Physiotherapist lacks ability in selecting questionnaires. | - Patient information - Symptoms of the patient |
| - 1. Interpreting the questionnaire results | - Physiotherapist reviews and interprets questionnaire results. - Physiotherapist integrates findings into the subjective examination. | - Physiotherapist lacks ability in assessing questionnaires. | - Diagnostic significance of the information |
| 1. Subjective examination of the patient | - Physiotherapist conducts an anamnesis interview about the patient's symptoms, medical history, and background. - Physiotherapist records and interprets the information to generate diagnostic hypotheses. | - Physiotherapist lacks ability in conducting history interviews. - Communicative difficulties between physiotherapist and patient. - Patient lacks compliance. | - Relevant/conspicuous information from the patient questionnaires - Main problem of the patient - Symptoms and pain - Symptom/pain localization - Symptom behavior/24h behavior - Short and long-term history - Previous and secondary diseases - Previous therapies - Contributing factors |
| - 1. Deciding which questions to ask the patient | - Physiotherapist inquiries about alarming symptoms to rule out serious diagnoses. | - Physiotherapist asks too few or avoids targeted diagnostic questions. | - Symptoms and pain of the patient - Red/yellow flags |
| - 1. Interpreting the information obtained | - Physiotherapist interprets information based on clinical patterns. | - Physiotherapist lacks knowledge in risk factors and clinical patterns. - Physiotherapist rejects information that doesn't fit the clinical pattern. | - Diagnostic significance of the information - Clinical patterns/disease patterns |
| - 1. Deciding when to stop eliciting information | - Physiotherapist stops the anamnesis interview once a satisfactory explanation is found. | - Physiotherapist forms opinions too early. | - Diagnostic significance of the information - Clinical patterns/disease patterns |
| - 1. Generating diagnostic hypotheses | - Physiotherapist generates diagnostic hypotheses early, based on questionnaire results and subjective findings. | - Physiotherapist focuses too much on one hypothesis. | - Relevant/conspicuous information from the patient questionnaires and the subjective examination |
| 1. Testing diagnostic hypotheses | - Physiotherapist tests hypotheses by examining relevant structures and functions. | - Physiotherapist struggles to integrate new, inconsistent information. - Patient lacks compliance. | - Diagnostic hypotheses - Patient information - Patient symptoms and pain - Physical and functional examination - Diagnostic tests |
| - 1. Deciding whether and which clinical examinations are to be carried out/requested | - Physiotherapist conducts clinical exams, uses diagnostic tests, trial treatments, and requests further tests if needed. | - Physiotherapist lacks ability in selecting/performing tests. - Physiotherapist conducts inadequate clinical examinations. | - Diagnostic hypotheses - Symptoms and pain of the patient |
| - 1. Interpreting the information obtained | - Physiotherapist interprets information according to the working hypothesis. | - Physiotherapist lacks experience in interpreting results. - Physiotherapist rejects information inconsistent with the leading working hypothesis. | - Diagnostic hypotheses - Diagnostic significance of the information |
| - 1. Deciding when to stop eliciting information | - Physiotherapist stops the clinical exam once hypotheses are confirmed, and alternatives checked. | - Physiotherapist conducts too few or inappropriate tests or forms opinions too early. | - Diagnostic hypotheses - Diagnostic significance of the information |
| 1. Consolidating and evaluating the information relevant to therapy | - Physiotherapist assesses questionnaire and exam results to form an overall picture. | - Physiotherapist lacks experience in interpreting information. | - Diagnostic hypotheses - Diagnostic significance of the information |
| - 1. Formulating the physiotherapeutic diagnosis | - Physiotherapist formulates a diagnosis based on therapy-relevant information and confirmed hypotheses. | - Physiotherapist lacks ability in choosing the physiotherapeutic diagnosis. - Physiotherapist misdiagnoses due to incorrect information or hypotheses. | - Therapy-relevant information - Appropriate diagnostic hypotheses |
| - 1. Creating a report of findings | - Physiotherapist compiles therapy-relevant information into a findings report. | - Physiotherapist chooses non-therapeutic information. | - Therapy-relevant information - Physiotherapeutic diagnosis |
| 1. Planning the adapted therapy | - Physiotherapist plans customized therapy, defining goals with the patient and creating a treatment plan. | - Inappropriate management due to misdiagnosis or insufficient knowledge. | - Physiotherapeutic diagnosis - Therapy-relevant information |
| - 1. Defining therapy goals | - Physiotherapist asks about patient goals and defines therapy goals together. | - Inappropriate treatment goals due to misdiagnosis or patient goals. | - Physiotherapeutic diagnosis - Therapy-relevant information - Patient goals |
| - 1. Creating a treatment plan | - Physiotherapist develops an initial treatment plan using evidence-based interventions and clinical guidelines. | - Physiotherapist lacks knowledge of evidence-based interventions and guidelines. | - Physiotherapeutic diagnosis - Therapy goals - Evidence-based interventions - Clinical guidelines |
| 1. Treating a patient's specific problem *(iterative approach)* | - Physiotherapist plans further treatment based on findings, diagnosis, and therapy goals. | - Inappropriate management due to misdiagnosis or insufficient knowledge. | - Physiotherapeutic diagnosis - Therapy goals - Therapy-relevant information from basic findings and repeat findings |
| - 1. Deciding whether and which clinical examinations are to be carried out/repeated | - Physiotherapist reassesses therapy-relevant information using the same diagnostic tests. | - Physiotherapist lacks ability in selecting or performing tests. - Physiotherapist performs inadequate reassessments. | - Current and past examinations - Relevant diagnostic tests |
| - 1. Interpreting the information obtained | - Physiotherapist compares re-evaluation results with initial findings to determine long-term treatment effects. | - Physiotherapist lacks experience in interpreting test results and treatment effects. - Physiotherapist rejects information inconsistent with diagnosis or treatment plan. | - Results of the relevant diagnostic tests from the basic findings - Long-term effect of the treatment |
| - 1. Deciding which body structures to treat | - Physiotherapist screens affected and possible related structures. | - Physiotherapist lacks ability in screening or trial treatments. | - Symptoms and pain of the patient - Abnormal body structures |
| - 1. Deciding which interventions to carry out | - Physiotherapist implements evidence-based interventions following clinical guidelines. | - Physiotherapist lacks knowledge of evidence-based interventions and clinical guidelines. | - Abnormal body structures - Evidence-based interventions - Clinical guidelines |
| - 1. Deciding whether to adapt the treatment | - Physiotherapist checks short-term treatment effects and adjusts if needed. | - Physiotherapist lacks experience interpreting treatment results. | - Short-term effect of the treatment |
| - 1. Deciding whether to stop treatment | - Physiotherapist checks long-term treatment effects and therapy goals; stops treatment if goals are achieved. | - Physiotherapist lacks experience interpreting treatment results. | - Long-term effect of the treatment - Therapy goals |
| 1. Evaluating the therapy results | - Physiotherapist compares current condition with pre-treatment condition and identifies influencing factors. | - Physiotherapist lacks experience to interpret therapy outcomes or self-reflect. | - Physiotherapeutic diagnosis - Course of treatment - Pre-post comparison of relevant information |
| - 1. Deciding which patient questionnaires and diagnostic tests to repeat | - Physiotherapist conducts a final reassessment using the same questionnaires and tests. | - Patient provides incomplete or untruthful answers. - Physiotherapist lacks ability in selecting or performing tests. - Physiotherapist conducts an inadequate final assessment. | - Relevant patient questionnaires - Relevant examinations from the survey |
| - 1. Deciding whether the therapy was successful and effective | - Physiotherapist compares current and original information to check if therapy objectives and patient goals are achieved. | - Physiotherapist lacks ability in assessing questionnaires and calculating scores. - Physiotherapist lacks experience interpreting test results. - Physiotherapist rejects information that doesn't meet expectations. | - Relevant information from the patient questionnaires and the subjective and clinical examinations - Patient and therapy goals |
| - 1. Checking patient satisfaction | - Physiotherapist asks about patient satisfaction with therapy and condition. | - Patient has unreasonable expectations about therapy results. | - Patient details - Patient goals |
