# Supplementary material for: Identifying Design Requirements for an Interactive Physiotherapy Dashboard With Decision Support for Clinical Movement Analysis of Musicians With Musculoskeletal Problems: Qualitative User Research Study
Source: JMIR Hum Factors. 2025 Jul 16;12:e65029. doi: 10.2196/65029 (PMC12292032; doi:10.2196/65029)
Supplement: Multimedia Appendix 3 [file humanfactors-v12-e65029-s003.docx]

# Multimedia Appendix 3: Key decision requirements (DR), associated information needs and design seeds (S).

| **Decision Requirement (DR)** | | **Information needs** | **Design seeds (S)** | |
| --- | --- | --- | --- | --- |
| **D_R1_** | **Plan functional diagnostic examination** *(Deciding whether and how an CMA is to be carried out)* | | | |
| D_R1-1_ | Decide whether and which examination is requested | - Patient information - Contributing factors - Symptom areas of the patient - Previous examination parameters | **S_1-1_**  **S_1-2_** | Note if musician-specific CMA is possible  Suggestion of examination parameters |
| D_R1-2_ | Decide when an examination is requested | - Number and timing of treatment units | **S_1-3_**  **S_1-4_** | Note on pre-interventional examination  Note on post-interventional examination |
| **D_R2_** | **Assess findings** *(Deciding how to interpret the results of the findings and how to proceed further)* | | | |
| D_R2-1_ | Gain an overview of a patient's problems | - Results of the respective individual findings   - Questionnaires   - Anamnesis   - Physical examination   - Functional examination - Possible comparative and reference values | **S_2-1_**  **S_2-2_**  **S_2-3_**  **S_2-4_**  **S_2-5_** | Presenting essential information in an aggregated and organized view  Organize the information in lists, tables, charts, and figures  Provide an interactive "drill down" function to view details of an entry  Provide adequate displays to enable efficient inspection of features  Provide more configurable displays to choose an appropriate presentation of the features |
| D_R2-2_ | Identify dysfunctional movement patterns and postures specific to different instruments | - See above | **S_2-6_**  **S_2-7_**  **S_2-8_**  **S_2-9_**  **S_2-10_**  **S_2-11_**  **S_2-12_** | Use of time-based displays to show time-dependent data over time  Use of comparative displays to enable comparison of data and characteristics  Use visual cues to draw attention to important and salient data and information  Integrating and presenting comparative and reference values  Providing tools and displays to select appropriate forms of presentation of different characteristics  Providing tools to investigate relationships between different characteristics  Providing an interactive function to mark/highlight relevant entries and characteristics |
| D_R2-3_ | Test diagnostic hypotheses | - Working hypotheses - Important and conspicuous data and information (see above) | **S_2-13_**  **S_2-14_**  **S_2-15_** | Provide an interactive function to assign entries/characteristics to a hypothesis  Provide a display to show entries/characteristics that have been assigned to a hypothesis  Provide an interactive function to flag applicable or non-applicable hypotheses |
| D_R2-4_ | Formulate a physiotherapeutic diagnosis | - Confirmed diagnostic hypotheses (see above) | **S_2-16_** | Provide a display to show applicable and inapplicable hypotheses |
| **D_R3_** | **Prepare overall finding and, if necessary, report of findings** *(Deciding which information is relevant to treatment and should be added to the overall report and how it should be presented)* | | | |
| D_R3-1_ | Select information relevant to therapy | - Physiotherapeutic diagnosis - Patient information - Data and information of the respective individual findings (see above) | **S_3-1_**  **S_3-2_**  **S_3-3_**  **S_3-4_** | Providing tools to efficiently create an overall report  Providing tools to efficiently create a report of findings  Providing an interactive function to efficiently add or remove information from the individual findings  Providing a configurable search and filter function |
| D_R3-2_ | Select forms of presentation | - See above | **S_3-5_** | Provide tools and displays to select appropriate display formats for different characteristics |
| **D_R4_** | **Compare results of reassessments and evaluate treatment effectiveness.** *(Deciding whether the treatment has a positive effect on the patient's problem or whether it needs to be adapted)* | | | |
| D_R4-1_ | Check therapy-relevant information before reassessment | - Therapy-relevant data and information from the baseline findings and, if applicable, the previous findings | **S_4-1_**  **S_4-2_**  **S_4-3_**  **S_4-4_**  **S_4-5_**  **S_4-6_**  **S_4-7_** | Presenting essential information in an aggregated and organized single view  Organize information in lists, tables, charts, and figures  Provide an interactive "drill-down" function to view details of an entry  Provide adequate displays to enable efficient inspection of features  Provide configurable displays to select an appropriate presentation form for the features  Providing a configurable search and filter function  Mapping of therapy events on a timeline |
| D_R4-2_ | Identify trends and changes after the reassessments or before and after treatment | - Therapy-relevant data and information from the baseline findings, any previous findings, and the current reassessment - Treatments and treatment results - Possible comparative and reference values | **S_4-8_**  **S_4-9_**  **S_4-10_**  **S_4-11_**  **S_4-12_**  **S_4-13_**  **S_4-14_** | Presenting key treatment outcomes (e.g., improvement/worsening of pain or function)  Use of time-based displays to show trends and highlight abnormalities  Use of comparative displays to enable comparison of characteristics  Use visual cues to draw attention to important and salient data and information  Integrating and presenting standard and comparative values  Providing tools to investigate relationships between different characteristics  Providing an interactive function to mark/highlight relevant entries and characteristics |
| **D_R5_** | **Evaluate therapy results and success** *(Deciding whether the therapy was successful and which factors were essential for this)* | | | |
| D_R5-1_ | Identify trends and changes following the final findings (last reassessment) | - Therapy-relevant data and information from the baseline findings and, previous findings - Treatment entries and results - Post-interventional results of relevant patient questionnaires, if applicable - Post-interventional results of the CMA, if applicable - Comparison and reference values | **S_5-1_**  **S_5-2_**  **S_5-3_**  **S_5-4_**  **S_5-5_**  **S_5-6_**  **S_5-7_**  **S_5-8_**  **S_5-9_** | Visualize key treatment outcomes (e.g., improvement/deterioration of pain or function)  Use of time-based displays to show relevant events on a timeline  Use of time-based displays to show trends and highlight anomalies  Use of comparative displays to enable a comparison of characteristics  Use of comparative displays to enable a pre-post comparison of features  Use of visual cues to draw attention to important and conspicuous data and information  Integrating and displaying standard and comparative values  Provide tools to investigate correlations between different characteristics  Providing an interactive function to mark/highlight relevant entries and features |
| D_R5-2_ | Check the therapy and patient goals | - Therapy and patient goals | **S_5-10_** | Provide an interactive function to check the achievement or non-achievement of a goal |
